# Epicardial fat volume is associated with coronary endothelium-dependent vasomotor response in healthy subjects

**DOI:** 10.1186/1532-429X-13-S1-P71

**Published:** 2011-02-02

**Authors:** Bénédicte Gaborit, Pierre Julien Moro, Antonin Flavian, Frank Kober, Alexis Jacquier, Jacques Quilici, Thomas Cuisset, Umberto Simeoni, Patrick Cozzone, Monique Bernard, Anne Dutour

**Affiliations:** 1 INSERM U626, Marseille, F-13385 France , Centre de Résonance Magnétique Biologique et Médicale (CRMBM), CNRS UMR 6612, Department of Endocrinology, Metabolic Diseases and Nutrition, CHU Nord, Marseille, France, Marseille, France; 2 Centre de Résonance Magnétique Biologique et Médicale, CNRS UMR N°6612,, Marseille, France; 3 Centre de Résonance Magnétique Biologique et Médicale (CRMBM), CNRS UMR 6612,, Marseille, France; 4 Department of Cardiology, CHU Timone, Marseille, France, Marseille, France; 5 INSERM U626, Marseille, F-13385 France , Department of Cardiology, CHU Timone, Marseille, France, Marseille, France; 6 Department of Neonatalogy, Children and Parents Pole, CHU Timone, Marseille, France, Marseille, France

## Introduction

Epicardial fat (E_fat_) is an active ectopic fat depot, directly surrounding coronary arteries, and secreting high level of inflammatory adipokines; its development has been associated with coronary atherosclerosis. We investigated the relationship between E_fat_ and endothelium dependent vasoreactivity of the coronary microcirculation.

## Methods

Myocardial blood flow (MBF) was determined by measuring coronary sinus flow with velocity-encoded cine magnetic resonance imaging at 3 teslas. We measured MBF at baseline and in response to sympathetic stimulation by cold pressor testing (CPT) in 17 healthy volunteers with normal left ventricular function (age 24±6 years, BMI=21.1±2.6kg/m2). E_fat_ volume was volumetrically assessed by manual delineation on short-axis views. CPT was applied by immersing one foot in ice water for 4 minutes.

## Results

A significant increase in MBF was observed: 1.18±0.58 vs 0.84±0.47mL.min-1.g-1, CPT vs rest, p=0.002. Mean relative MBF increase (ΔMBF) was 50±47%. Mean E_fat_ volume was 82±31mL and varied from 43 to 131 mL; mean LV mass and Left ventricular ejection fraction were 104±31g and 64±5%, respectively. CPT significantly increased heart rate (HR) by 28±13%, systolic blood pressure (BP) by 17±13%, diastolic BP by 23±19% and rate-pressure product by 52±25%, p<0.01, indicating an increase in myocardial work load. The increase in HR, reflecting sympathetic stimulation, was not influenced by sex, age or E_fat_ volume. CPT induced a decrease in coronary vascular resistance (150±93 vs 114±44 mmHg.mL-1.min.g) by trend (p=0.08). Interestingly, we found a significant negative correlation between E_fat_ volume and ΔMBF (r=-0.51, p=0.03), which remained significant after adjusting for age and sex. ΔMBF was not associated with waist circumference, BMI, CRP, lipid or glycemic parameters.

## Conclusion

The increase in E_fat_ is associated with a decrease in endothelium dependent vasoreactivity response, suggesting that E_fat_ could early influence endothelial function.

